# Impact of a patient-centered medical home demonstration on quality of life and patient activation for older adults with complex needs in Singapore

**DOI:** 10.1186/s12877-021-02371-y

**Published:** 2021-07-23

**Authors:** Grace Sum, Soon Hoe Ho, Zoe Zon Be Lim, Junxing Chay, Mimaika Luluina Ginting, Mary Ann Tsao, Chek Hooi Wong

**Affiliations:** 1 Geriatric Education and Research Institute, Singapore, Singapore; 2 Lien Centre for Palliative Care, Duke-NUS Medical School, Singapore, Singapore; 3 Tsao Foundation, Singapore, Singapore; 4 Health Services & Systems Research, Duke-NUS, Singapore, Singapore

**Keywords:** Patient-centered medical home, Patient-centered care, Older adults, Geriatrics, Quality of life, Patient activation

## Abstract

**Background:**

The first Patient-Centered Medical Home (PCMH) demonstration in Singapore was launched in November 2016, which aimed to deliver integrated and patient-centered care for patients with bio-psycho-social needs. Implementation was guided by principles of comprehensiveness, coordinated care, shared decision-making, accessible services, and quality and safety. We aimed to investigate the impact of implementing the PCMH in primary care on quality of life (QoL) and patient activation.

**Methods:**

The study design was a prospective single-arm pre-post study. We applied the 5-level EuroQol 5-dimension (EQ-5D-5L) and Visual Analog Scale (EQ VAS) instruments to assess health-related QoL. The CASP-19 tool was utilised to examine the degree that needs satisfaction was fulfilled in the domains of Control, Autonomy, Self-realisation, and Pleasure. The 13-item Patient Activation Measure (PAM-13) was used to evaluate knowledge, skills and confidence in management of conditions and ability to self-care. Multivariable linear regression models with random intercepts were applied to examine the impact of the PCMH intervention on outcome measures at 3 months and 6 months post-enrolment, compared to baseline.

**Results:**

We analysed 165 study participants enrolled into the PCMH from November 2017 to April 2020, with mean age 77 years (SD: 9.9). Within-group pre-post (6 months) EQ-5D-5L Index (β= -0.01, *p*-value = 0.35) and EQ VAS score (β=-0.03, *p*-value = 0.99) had no change.

Compared to baseline, there were improvements in CASP-19 total score at 3 months (β = 1.34, *p*-value = 0.05) and 6 months post-enrolment (β = 1.15, *p*-value = 0.08) that were marginally out of statistical significance. There was also a significant impact of the PCMH on the CASP-19 Pleasure domain (β = 0.62, *p* = 0.03) at 6 months post-enrolment, compared to baseline. We found improved patient activation from a 15.2 % reduction in the proportion of participants in lower PAM levels, and a 23.4 and 16.7 % rise in proportion for higher PAM levels 3 and 4, respectively, from 3 months to 6 months post-enrolment.

**Conclusions:**

Preliminary demonstration of the PCMH model shows evidence of improved needs satisfaction and patient activation, with potential to have a greater impact after a longer intervention duration.

## Introduction

The global ageing population has posed an increased pressure on health and social care systems by adults with complex needs, including multiple chronic conditions, medication-related issues like polypharmacy and adverse drug reactions, and social vulnerability [1, 2]. Patients with a complex interaction of biology, psychological and social needs have a higher tendency of having poorer physical and mental health-related quality of life (QoL), worse depression severity, higher healthcare costs, and unmet health and social demands [3, 4]. There has also been recent recognition amongst primary care practitioners of the growing challenges of providing high quality and appropriate care for complex needs patients in several high-income nations [5]. Importantly, the literature has suggested a paradigm shift from a biomedical and disease-specific approach to a more comprehensive bio-psycho-social model that emphasises the interplay of physical illnesses, mental disorders, and social and home environmental problems [6]. This bio-psycho-social perspective promotes the application of treatment options that considers the unique preferences and goals of each patient, participatory clinician-patient relationships, and tools to improve patient engagement and QoL [7].

One concept that aims to address complex needs of patients is person-centered care (PCC), defined as “a partnership among practitioners, patients and their families to ensure that decisions respect patients’ wants, needs and preferences, and that patients have the education and support they require to make decisions and participate in their own care” [8]. PCC has been established as one of the six elements of quality of care by the Institute of Medicine (United States) Committee on Quality of Health Care in America, and a central component to modern health service delivery [9]. While PCC was more commonly applied in the setting of nursing homes and assisted-living facilities to improve normalisation of daily living in institutionalised older adults, there has been more emphasis on extending PCC to provide home- and community-based services (HCBS) for community-dwelling patients [10]. The Patient-Centered Medical Home (PCMH) is a model of care that reflects this shift in PCC to the community, as well as a shift from episodic primary care to delivery of high quality primary care, to increase the value of healthcare to whole persons, families, communities and populations [11]. The model reinforces core attributes of primary care and is guided by the principles of first contact accessibility, comprehensiveness and whole-person orientation, integration and care coordination, sustained healthcare professional-patient relationships, and quality and safety [11, 12].

The PCMH model has been associated with reduced healthcare utilisation and cost, better patient experiences, lowered clinician burnout, and improved care quality [13–16]. Additionally, the few existing studies on PCMH have focused on the effects on subgroups of patients like those with specific single non-communicable conditions or multimorbidity, who may or may not have complex needs, and have revealed mixed findings [17–20]. However, there is still a dearth in studies on how the PCMH model impacts patient-reported outcome measures, which are widely utilised in health services research to evaluate the effectiveness of care models and quality of healthcare delivery [21–23]. Care elements like provider-patient communication, self-management, and team-based care have been independently correlated with better QoL in multimorbid patients,[19] but the impact of these aspects combined on health-related QoL, needs satisfaction, and patient knowledge, skills and confidence have not been assessed for those with complex needs.

In Singapore, there are existing advanced primary care models, such as the Teamlet Care Model that focuses on chronic disease management by a multidisciplinary team, and Family Medicine Clinics (FMCs) where general practitioners provide individualised and targeted care at a one-stop platform for patients with chronic conditions [24, 25]. This PCMH model in this study goes beyond this by targeting community-dwelling older adults with complex bio-psycho-social needs based on medical, psychological, and social health screening and/or assessments, situated within a defined geographical region of Whampoa, Singapore. The PCMH model also emphasises empanelment of patients to provide high quality primary care for an extended period by a dedicated multidisciplinary care team. Importantly, by being embedded in this integrated ecosystem, the PCMH model of care abided by four overarching principles, including ageing in place (supporting older adults in growing old in their homes), life-course approach (promoting the earlier implementation of interventions for health in older persons), socio-ecological model of care (recognising health as an outcome of an individual’s interaction with family, caregivers, communities and society), and population health management.

Our study aims to evaluate how the PCMH model of care in Singapore would impact QoL and patient activation in community-dwelling older patients with complex needs. The PCMH in this study will be a model of care that consists of two integrated elements: (1) medical care in the primary care clinic and (2) psychosocial care in home-based care management. Implementation is guided by PCMH principles on patient-centeredness, comprehensiveness care, coordinated care, accessible services, shared decision-making, and quality and safety [12]. The PCMH is part of a larger integrated care initiative in Singapore, the Community for Successful Ageing (ComSA) by Tsao Foundation, a community-wide project on an integrated system of comprehensive programs and services aimed to promote the health and well-being of older adults [26]. Findings from this investigation would have important implications on changes in the delivery of primary healthcare and health policies for older adults with complex needs. This study also addresses the knowledge gap in implementing a PCMH model in an Asian context [27]. We hypothesise that older persons identified as having bio-psycho-social needs who receive the PCMH intervention would have improved self-reported QoL and patient activation over time.

## Methods

### Study design and setting

The study design is a prospective single-arm pre-post study. This study was registered with ClinicalTrials.gov (Protocol ID: 2017/00352), and all methods were performed in accordance with the relevant guidelines and regulations of the study registration submission. This study is part of an evaluation of a Patient-Centered Medical Home (PCMH) demonstration in Singapore, using multi-methods with concurrent quantitative and qualitative components. Findings on the qualitative perspectives of healthcare professionals, patients and caregivers will be published separately.

### Study participants

Study participants were recruited from 1 to 2017 to 30 April 2019. Informed consent was taken from participants or proxies. This study was approved by the National Healthcare Group Domain Specific Review Board (NHG DSRB) Singapore (Reference: DSRB 2017/00352). Intervention baseline was defined as the date first study survey was conducted, and the first study survey was conducted within the first 60 days from study enrolment date. Two follow-up surveys were conducted at 3 months and 6 months post-enrolment.

Eligibility criteria of study participants:

(i) Aged 40 years and This cut-off age was chosen to reflect the life course approach, whereby PCMH services could also be provided to patients with complex needs from their fourth decade of life, prior to entering old age, for early intervention to prevent further adverse health outcomes [28].
(ii) Had high bio-psycho-social health risk as defined by a 37-item Bio-Psycho-Social Risk Screener validated in the local setting, [29] or pre-existing risk stratification criteria used by referring healthcare institutes and/or clinical judgement.

The BPS Risk Screener is a locally validated survey instrument designed to be evaluated by trained surveyors, who do not need to be clinical psychologists [29]. The BPS Risk Screener is 37-item assessment that contains 19 questions on biological health (seeing/hearing/communication, looking after yourself, getting around), 12 questions on psychological health (feeling safe, emotional well-being), and 6 questions on social health (relationships, social independence). Each of the three domains contribute a score of 0, 1 or 2, whereby the higher the score, the greater the risk of poor health in that domain. The summation of scores ranges from 0 to 6, whereby scores 0 to 1 are categorised as “Managing well”, scores 2 to 3 are categorised as “Some problems”, scores 4 to 5 are categorised as “Many problems” and score 6 is categorised as “Overwhelming problems”. Patients with high bio-psycho-social health risk were those in the latter two categories.

(iii) Resided in Whampoa, a geographically defined district in Singapore (total population of 41,000) where the PCMH was located, and (iv) took first study survey within 60 days of enrolment into the programme.

### Intervention

**Fig. 1:**
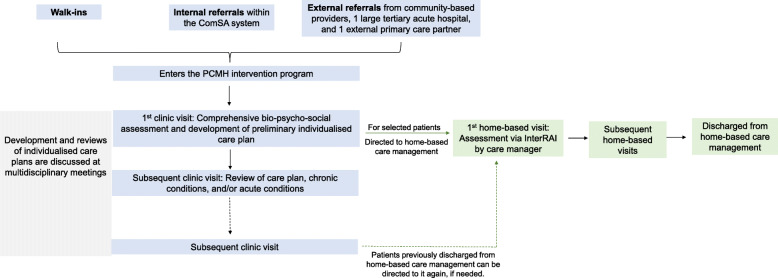
The patient-centered medical home program workflow. ComSA: Community for successful ageing; InterRAI: International resident assessment instrument home care

Figure 1. shows the PCMH program workflow. The PCMH is an integrated care intervention comprising (1) physician-led primary care clinic, and (2) medical social worker- and nurse-led home-based care management services. The intervention involved a multidisciplinary care team that consisted of a core team from the PCMH (includes physician, registered nurse, program coordinator and care managers (nurse, social worker, assistant care manager)), comprehensive needs assessment, and individualised care plans. The intervention aimed to meet patients’ complex bio-psycho-social needs by providing continuous, comprehensive and coordinated health and social care by the same care team.

Patients entered the PCMH program via walk-ins, internal referrals within the ComSA system, and external referrals from community-based providers, a large tertiary acute hospital, and a public primary care partner (Fig. 1). Referrals from the tertiary hospital were conducted through a program whereby geriatric medicine and internal medicine specialist clinics triaged patients based on an additional assessment on psycho-social needs. Primary care physicians of the PCMH were trained in the care of older adults and basic care coordination supported by registered nurses. The first clinic visit usually lasted for one hour due to the need for comprehensive bio-psycho-social assessment and development of a preliminary care plan with the patient and family members. The comprehensive assessment included taking medical history, management of acute health conditions, assessment for acute, sub-acute and chronic medical conditions, screening for mood, cognition and psycho-social issues, and a medication review. Nurses also assessed acute conditions, self-care capability, and functional needs.

Subsequent clinic visits were reviews of care plans and chronic conditions, or to treat acute conditions. Patients had individualised care plans based on needs, preferences and expectations, and family members and caregivers were involved in shared decision-making for care planning usually by attending the clinic visits together with patients. Different opinions and preferences between patients and their caregivers or family members were respected and discussed with care managers and healthcare providers. As the PCMH model aimed to have relationship-based care, care managers and healthcare providers sought to reach consensus with patients and their caregivers and family on individualised care plans. The development of care plans and subsequent reviews were discussed at interdisciplinary team meetings. The PCMH primary care providers also partnered geriatric specialists from the tertiary acute hospital to provide shared care, and they communicated via face-to-face meetings, tele-consultations, and sharing of medical records. This includes referring patients with depressive symptoms to psychologists for specialist intervention, after psychological assessment via the Geriatric Depression Scale (GDS) by PCMH clinic nurses.

Patients assessed to have complex bio-psycho-social needs at the initial assessment or follow-up visits at the clinic were directed to home-based care management services for further management. Care management aimed to extend care to the home setting, including the physical home environment, behavourial and financial needs, and support systems from family and caregivers. The first home visit, that usually has a duration of an hour, involved assessment using International Resident Assessment Instrument Home Care (interRAI-HC) by a trained care manager [30]. InterRAI-HC guided comprehensive care and service planning in the community-setting and provided quality assurance in monitoring patients’ progress [30]. The InterRAI-HC is a comprehensive assessment that assesses several domains, including socio-demographics, living arrangements, cognition, communication and vision, mood and behaviour, psychosocial well-being, functional status, locomotion and walking, continence, disease diagnoses, health conditions, pain symptoms, oral and nutritional status, skin conditions, medications, treatments and procedures, responsibilities and directives, social support network, environment assessment, and discharge potential and self-sufficiency [30]. The InterRAI-HC also contains questions contributing to an algorithm that generates a Depression Rating Scale (DRS) [30]. The responses are keyed into an InterRAI software program that generates results based on algorithms from decision support tools, and the results support the development of individualised care plans and provision of care management services. A nurse care manager conducted visits to check adherence to individualised care plans, a social worker care manager managed financial, and family and caregiver matters, and both the nurse and social worker care managers were responsible for assessing medication compliance, social engagement, and care coordination with services. Care plans were reviewed every six months to assess suitability for discharge. Patients previously discharged from care management services could be registered again when needed.

### Outcome measures

The survey was administered by trained surveyors, who conducted the surveys with study participants face-to-face using a hardcopy survey (i.e., paper and pen administration). In stances when proxies were required, the surveyors conducted the survey with proxies using the hardcopy proxy version survey. The survey questions were only surveyor-administered, and nurses or other persons did not assist.

#### EQ-5D-5L

The 5-level EuroQol 5-dimension (EQ-5D-5L) instrument was used to evaluate health-related QoL in the areas of mobility, self-care, usual activities, pain or discomfort, and anxiety or depression [31]. Each dimension was scored on a 5-point rating scale: no problems, slight problems, moderate problems, severe problems, and extreme problems [31]. Scores were combined and converted to single Index using the “EQ-5D-5L Crosswalk Index Value Calculator version 2.0” [32]. The EuroQol Visual Analog Scale (EQ VAS) was applied to obtain a quantitative measure of self-rated health on a scale of 0 to 100, with 0 being the worst possible health, and 100 the best health.

#### CASP-19

This study used the 19-item Control, Autonomy, Self-realisation, and Pleasure (CASP-19) tool specifically designed to assess QoL in older adults [33, 34]. The instrument was developed based on the theory of needs satisfaction, whereby quality of life in older adults was conceptualised as the degree to which human needs were satisfied in four life domains [33, 34]. Importantly, it is able to distinguish overall subjective quality of life from the contextual factors that influence it, such as health, finances, social support, and participation [34, 35]. The control domain examines an individual’s ability to actively control their environments [36]. The autonomy domain evaluates the freedom from unwanted interference of others [36]. Self-realisation and pleasure domains assess aspects of living that individuals derive reward and happiness in their later life [36]. There are 4-point rating scales for each item: Never, not often, sometimes, and often [37]. Scores for the Control domain (4 items) ranged from 0 to 12, and scores for the Autonomy, Pleasure and Self-realisation domains (5 items each) ranged from 0 to 15. Total score was derived by summing responses of all items (ranged from 0 to 57) [37]. The lower the score, the poorer the quality of life in later life.

#### PAM-13

The 13-item Patient Activation Measure (PAM-13) instrument evaluated patient knowledge, skills and confidence in their management of disease conditions and ability to self-care [38]. Insignia Health generated the activation score (ranged from 0 to 100) and level (ranged from 1 to 4) of each participant based on survey data [39]. The lower the score or level, the poorer the patient activation.

### Data analysis

Sample characteristics of the 165 participants analysed were presented, and data were summarised descriptively as mean and standard deviation.

We conducted multivariable linear regression models with random intercepts to examine the difference between mean scores at 3 months and 6 months post-enrolment, compared to baseline, adjusting for age at enrolment, sex, weighted Charlson Comorbidity Index (CCI) at baseline, having received any formal education (yes; no), housing types in Singapore (1-, 2- and 3-room Housing Development Board (HDB) apartments; 4-room or larger HDB apartments and Housing and Urban Development Company (HUDC) apartments and executive condominiums) and baseline PAM-13 score. These covariates were selected based on their plausible relationships with QoL and patient activation. Ethnicity was not adjusted for as Chinese were overrepresented in the sample. We used the weighted CCI as the summary measure for adjusting for comorbidities in our multivariable linear regression model. The CCI is based on the number of chronic conditions that are each assigned an integer weight from one to six, with a weight of six representing the most severe morbidity [40]. The summation of the weighted comorbidity scores results in a summary score [40]. In this study, the ICD-10 codes of study participants were based on a national healthcare administrative database and the PCMH clinic administrative database. Subsequently, we used the command from the statistical software to compute the weighted CCIs based on ICD-10 codes. This computed weighted CCI was a covariate in the multivariable regression model.

Cluster-robust standard errors were reported. Statistical significance was at *p* < 0.05. All analyses were performed on Stata v14.0 (StataCorp).

## Results

### Participant baseline characteristics

**Fig. 2:**
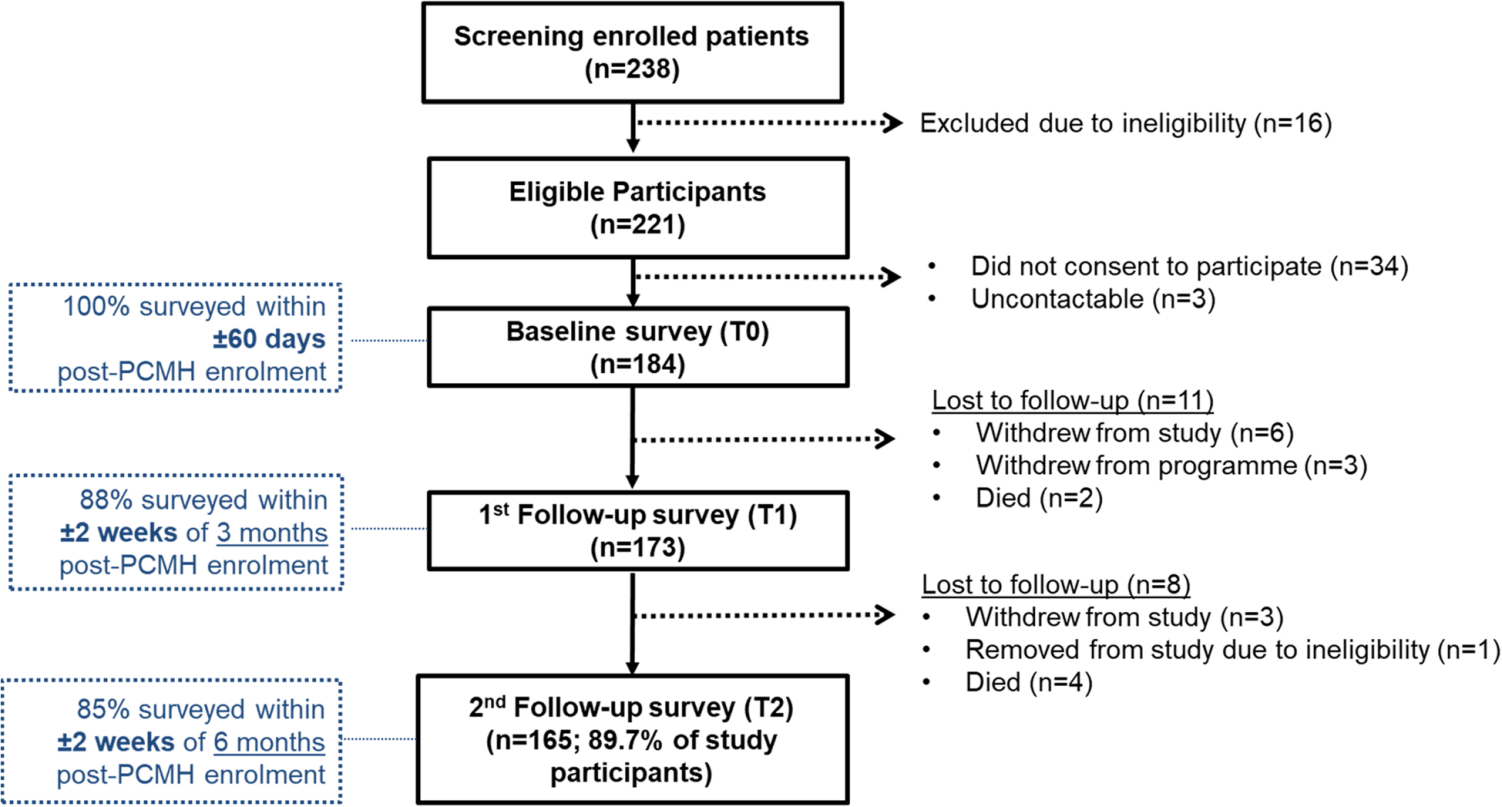
Study participant flow chart (N analysed = 165)

Figure 2 presents the study participant flow diagram and progression of the intervention from baseline to 6 months post-enrolment into the PCMH. A total of 238 patients were enrolled into the PCMH from 1 to 2017 to 30 April 2019, of which 16 did not fulfil study eligibility criteria. After excluding patients who did not consent to the study (*n* = 34 (14.3 %)) and were uncontactable (*n* = 3), this study recruited 184 study participants. The final sample size analysed was 165 study participants after a loss to follow up at 3 months post-enrolment (*n* = 11 (6.0 %)) and 6 months post-enrolment (*n* = 8 (4.3 %)). There were 6 deaths, 1 patient who was retrospectively found to be ineligible, and 12 withdrew from PCMH due to being home-bound, admitted to a long-term care facility, or relocated to be out of the PCMH service boundary.

Table 1. displays the sociodemographic baseline characteristics of study participants (*n* = 165). Mean age of study participants was 77 years, with 93.9 % aged 60 years and above. The proportion of males was 43.6 %, 51.5 % were married, a majority of 93.3 % were ethnic Chinese, 48.5 % had no formal education, and 58.8 % stayed in a smaller housing type. Study participants had a mean of 5 chronic conditions per person, based on self-report from a list of 17 chronic conditions (diabetes, heart conditions, hypertension, high cholesterol, incontinence (urinary or faecal), osteoporosis, stroke, neurological diseases (e.g. Parkinson’s, epilepsy), eyesight problems, hearing problems, arthritis/rheumatism/joint or nerve pain, back pain, chronic lung disease (asthma, chronic obstructive pulmonary disease), dementia, anxiety, depression, and frequent falls (≥ 2 falls in past 12 months).

**Table 1:** Sociodemographic characteristics of study participants

Sociodemographic characteristic	*n* = 165
**Age at enrolment, years, mean (SD)**	77.0 (9.88)
**Age group, years, n (%)**	
40–49 years	2 (1.21)
50–59 years	8 (4.85)
60–69 years	26 (15.76)
70–79 years	63 (38.18)
80–89 years	49 (29.70)
≥ 90 years	17 (10.30)
**Sex, n (%)**	
Male	72 (43.64)
Female	93 (56.36)
**Ethnicity, n (%)**	
Chinese	154 (93.33)
Malay	3 (1.82)
Indian	7 (4.24)
Others	1 (0.61)
**Marital status, n (%)**	
Single	14 (8.48)
Married	85 (51.52)
Widowed	53 (32.12)
Divorced	13 (7.88)
**Education, n (%)**	
No formal education	80 (48.48)
Primary school	51 (30.91)
Secondary school	23 (13.94)
Post-secondary (non-tertiary)	8 (4.85)
Diploma and professional	3 (1.82)
**Housing type, n (%)**	
**Smaller housing type**	
1–2 room HDB apartment	16 (9.70)
3-room HDB apartment	81 (49.09)
**Larger housing type**	
4-room HDB apartment	47 (28.48)
5-room HDB apartment, HUDC apartment, EC	20 (12.12)
Private condominium/private others	1 (0.61)
**Employment status, n (%)**	
Employed full-time	14 (8.48)
Employed part-time	13 (7.88)
Unemployed	7 (4.24)
Retired	127 (76.97)
Others	4 (2.42)
**Chronic disease status**	
Mean no. of chronic conditions	5.17
Weight CCI	4.82

*HDB* Housing Development Board; *HUDC* Housing and Urban Development Company; *EC* Executive Condominium; *CCI* Charlson Comorbidity Index; Chronic disease list: hypertension, high blood cholesterol, arthritis, eyesight problems, back pain, diabetes, hearing problems, incontinence, frequent falls, dementia, heart conditions, stroke, chronic lung disease, osteoporosis, depression, anxiety, neurological diseases, others

### Quality of life

The baseline EQ-5D-5L Index and EQ VAS score were 0.77 (SD: 0.18) and 70.25 (SD: 18.62), respectively. There were no significant differences in EQ-5D-5L Index values and EQ VAS scores at 3 months (EQ-5D-5L: β = 0.01, *p*-value = 0.16; EQ VAS: β= -0.94, *p*-value = 0.52) and 6 months post-enrolment (EQ-5D-5L: β= -0.01, *p*-value = 0.35; EQ VAS: β= -0.03, *p*-value = 0.99) into the PCMH intervention, compared to baseline (Table 2). Regarding the dimension on anxiety and depression of the EQ-5D-5L Index, the mean baseline score was 1.42 (SD: 0.83), and the mean scores at 3 months and 6 months post-enrolment were 1.28 (SD: 0.66) and 1.39 (SD: 0.77), respectively.

**Table 2:** Quality of life outcome measures (*n* = 165)

Outcome measure	Baseline	3 months post-enrolment	β1 (95 %CI)	6 months post-enrolment	β2 (95 %CI)
**Quality of life, mean (sd)**					
**EQ-5D-5L Index Value** (0 to 1)	0.77 (0.18)	0.78 (0.18)	0.01 (-0.004, 0.02)	0.76 (0.20)	-0.01 ( -0.02, 0.01)
**EQ VAS** (0 to 100)	70.25 (18.62)	69.41 (17.95)	-0.94 ( -3.79, 1.92)	70.29 (18.28)	-0.02 ( -3.02, 2.97)
**Quality of life in older adults, mean (sd)**					
**CASP-19 Total Score**	32.12 (10.75)	33.48 (11.25)	1.34 ( -0.01, 2.68)	33.31 (10.58)	1.15 (-0.14, 2.45)
**CASP-19 domain scores**					
Control	5.75 (3.22)	6.18 (3.32)	0.41 (-0.08, 0.89)	5.60 (3.02)	-0.16 (-0.59, 0.27)
Autonomy	10.47 (2.95)	10.47 (3.00)	-0.025 (-0.46, 0.41)	10.70 (2.89)	0.20 (-0.23, 0.63)
Self-realisation	6.40 (3.71)	6.91 (3.69)	0.52 (-0.04, 1.07)	6.89 (3.41)	0.50 (-0.05, 1.04)
Pleasure	9.50 (3.77)	9.92 (3.68)	0.44 ( -0.12, 1.00)	10.12 (3.66)	0.62 (0.079, 1.16)*
**Patient Activation, mean (sd)**					
**PAM-13 Total Score**	54.82 (10.32)	53.52 (8.0)	-1.25 ( -2.73, 0.23)	54.28 (9.29)	-0.53 ( -2.03, 0.97)

*p-value < 0.05

**p-value < 0.005

β1 represents the mean change in score from baseline to 3 months post-enrolment into the PCMH, and β2 represents the mean change in score from baseline to 6 months post enrolment into the PCMH adjusting for age at enrolment, sex, weighted Charlson Comorbidity Index (CCI) at baseline, having received any formal education (yes; no), housing types in Singapore (1- 2- and 3-room Housing Development Board (HDB) apartments; 4-room or larger HDB apartments and Housing and Urban Development Company (HUDC) apartments and executive condominiums) and baseline PAM-13 score

The baseline total score for CASP-19 was 32.12 (SD: 10.75), and the baseline domain scores for Control, Autonomy, Self-realisation, and Pleasure were 5.75 (SD: 3.22), 10.47 (SD: 2.95), 6.40 (SD: 3.71), and 9.50 (3.77), respectively. This study found improvements in the CASP-19 total score at 3 months (β = 1.34, *p*-value = 0.05) and 6 months (β = 1.15, *p*-value = 0.08) post-enrolment into the PCMH, compared to baseline, but these were marginally out of statistical significance. There were no significant differences in the domain scores for Control, Autonomy and Self-realisation at 3 months and 6 months post-enrolment, compared to baseline. However, the Pleasure domain score was significantly higher at 6 months post-enrolment (β = 0.62, *p* = 0.03), compared to baseline.

### Patient activation

The baseline total score for PAM-13 was 54.82 (SD: 10.32). There were no significant changes in the PAM-13 total score and proportions of participants in different PAM levels at 3 months and 6 months post-enrolment into the PCMH, compared to baseline (Table 2).

There was still some evidence a positive effect in the activation of participants to becoming more engaged in managing their health from 3 months to 6 months post-enrolment into the intervention, whereby the proportion of participants in the lower PAM levels, Level 1 (disengaged and overwhelmed) and Level 2 (Becoming aware but still struggling), dropped by 15.2 % (from 60.0 to 50.9 %). Correspondingly, the proportion of participants in the higher PAM Level 3 (Taking action) increased by 23.4 % (from 36.4 to 44.9 %), and Level 4 (Maintaining behaviours and pushing further) rose by 16.7 % (from 3.6 to 4.2 %) (Table 3).

**Table 3:** Patient activation levels (*n* = 165)

Outcome measure	Baseline	3 months post-enrolment	6 months post-enrolment
**PAM-13 Levels, n (%)**			
**Level 1** (disengaged and overwhelmed)	37 (22.4)	40 (24.2)	38 (23.0)
**Level 2** (becoming aware but still struggling)	54 (32.7)	59 (35.8)	46 (27.9)
**Level 3** (taking action)	64 (38.8)	60 (36.4)	74 (44.9)
**Level 4** (maintaining behaviours and pushing further)	10 (6.1)	6 (3.6)	7 (4.2)

Compared to baseline, proportions at 3 months and 6 months post-enrolment were not statistically significantly different via Chi-square (*χ*^2^) tests

## Discussion

Our study evaluated the impact of a community-based PCMH on QoL and patient activation in adults with complex bio-psycho-social needs in Singapore, using a prospective single-arm pre-post design. Although there were no significant differences in QoL measured by the EQ-5D-5L index, EQ VAS score, and CASP-19 total score, and patient activation assessed with the PAM-13 score, at 3 months and 6 months post-enrolment, this study still found evidence of positive effects of the PCMH intervention. Compared to baseline, there were improvements in CASP-19 total scores at 3 months (β = 1.34, *p*-value = 0.05) and 6 months (β = 1.15, p-value = 0.08) post-enrolment that were marginally out of statistical significance, and there was a significant impact of PCMH on the CASP-19 Pleasure domain (β = 0.62, *p* = 0.03) at 6 months post-enrolment. We found improved patient activation from a 15.2 % reduction in the proportion of participants in lower PAM levels and a 23.4 and 16.7 % rise in proportion for higher PAM levels 3 and 4, respectively, from 3 months to 6 months post-enrolment.

Comparison of our findings on QoL with current literature is challenging due to a limited number of studies applying a comparable PCMH care model that targeted complex needs patients. While improvements in QoL scores from the implementation of a PCMH care model have been reported, those studies focused on populations with specific single conditions like type-2 diabetes or low-income [17, 20]. In addition, existing studies examined patients with physical multimorbidity rather than bio-psycho-social needs, and reported mixed results on QoL. For instance, a randomised controlled trial on approximately 3,000 patients with type-2 diabetes, chronic obstructive pulmonary disease and/or chronic cardiovascular conditions and high likelihood of hospitalisation predicted by insurance data, reported that no significant difference in EQ-5D but better Short-Form-12 (SF-12) scores at 24 months post-intervention [18]. Comparison of our findings on the mental health (anxiety/depression) dimension score of EQ-5D-5L that remained unchanged post-intervention is difficult due to the lack of studies on a similar PCMH model. However, there is still some evidence that shows that the mental health component also remains unchanged from PCMH, such as a recent retrospective cohort study in the United States by Schuttner et al. (2020) that examined the effect of PCMH on approximately 22,000 patients with multimorbidity, and revealed no significant differences in the mental component scores of the SF-12 survey, but physical component scores improved [19].

Our finding on improved CASP-19 scores have implications on PCMH having potential effects on the contextual influences on quality of life in early old age, such as social support and participation, health and financial security, and feelings of trust and reciprocity in their environments [41]. While EQ-5D-5L and EQ VAS tools examine health status, CASP-19 evaluates aspects of life and needs satisfaction not defined by health [33, 37, 41]. The CASP-19 assesses active and reflexive areas of living, instead of the absence of poor health and securing basic day-to-day needs [33, 42]. Additionally, a systematic review by Bulamu et al. (2015) on instruments for assessing QoL in older persons proposes that the newer CASP-19 instrument may be more appropriate to capture broader dimensions of quality of life for older persons, compared to more generic tools like the EQ-5D [43]. Our application of CASP-19 is a reflection of the complex health (mean age of 77 years, high mean number of 5 chronic conditions and CCI) and social needs (majority with no formal education and resides in a smaller housing type) of this study’s participants. Importantly, this study provides some justification for further exploration of patients’ inherent needs and reflexive areas of living beyond the absence of physical and mental health conditions [44].

Comparison of our study findings is limited by the dearth in literature that examines the effects of integrated care models on CASP-19, with most studies on the evaluation of the tool, [42, 45] or PCMH contextual factors that impact specific subpopulations [46, 47]. However, the mean total CASP-19 score in this study at 6 months post-enrolment into the PCMH is lower at 33.3, as compared to cross-sectional studies in England on a nationally representative sample of 9,300 non-institutionalised adults with mean age 65 years and in Taiwan on 6,009 elderly during a hospital-based annual health examination, which reported mean total scores of 42.5 and 38.2, respectively [35, 48]. A likely explanation is that the cross-sectional studies did not select for complex needs patients, and this further proves that our study targeted patients with greater bio-psycho-social needs than the general elderly population. While only the Pleasure domain, which represents the “degree of fun” that an individual experienced in doing things in life, [37] had significant improvement from the PCMH, we hypothesise that the other domains may show improvements with more time in the intervention and a larger sample size.

Our finding on the shift in proportion of participants from lower to higher PAM levels is important as studies reveal that higher PAM activation stages are positively associated with higher functional status, adherence to health behaviours, better clinical indicators, use of preventive screening tests, and lower costs to health systems [49–51]. This shift may reflect the change from being passive recipients of care, to having more proactive engagement in recommended health behaviours [46, 47, 51]. Measuring PAM levels could be an important aspect in PCMH to tailor components of care education based on each patient’s activation level, and act as an intermediate measure of patient care outcomes and feedback for the multidisciplinary team [52]. Our study may suggest that the component of home-based care management in the PCMH model may strengthen psychosocial care for the patients, and promotes more enjoyment in life through social interaction and participation in new activities and interests [37, 41, 53].

Strengths of this study include the PCMH program being a complex intervention that contained multiple components for meeting the multi-dimensional needs of patients, including aspects of comprehensive assessments in the clinic and the home setting, a multidisciplinary care team, individualised care plans, shared decision-making as well as empanelment, having strict inclusion criteria for study participants, a low loss to follow up rate, and using validated instruments to examine outcomes. Our study contributes to the literature by investigating the impact of PCMH on quantitative patient-reported outcome measures in an Asian context, with current literature in Singapore focusing on qualitatively examining implementation challenges [27].

However, the study had a few limitations. The short duration of intervention of 6 months and sample size possibly led to the inability to yield differences in QoL and patient activation measures of statistical significance. Power calculations for the study were based on 80 % power to detect a 10 % reduction in inpatient utilisation, instead of changes in QoL and patient activation measures. Additionally, the baseline EQ-5D-5L Index, EQ VAS score, CASP-19 score and PAM-13 score were not considered to be at high end of the spectrum, so there was potential for these study participants with complex needs to have improved outcome measures. Hence, the authors suggest that the shorter duration and sample size were more likely factors in the lack of statistical significance, and acknowledge that the implications drawn from this study need to account for the lack of statistical significance in some results. Next, randomisation was not feasible for this study, which limited our study design to a within-group prospective before-after evaluation without a comparator. While this study did not have a control group, it still contributed to showing that complex intervention with health and social integration in primary care could improve needs satisfaction and patient activation. We hypothesis that our study is biased towards the null, whereby a randomised control trial would yield greater benefits from the PCMH as high-risk controls would likely demonstrate decreased QoL and poorer health outcomes. In addition, a limitation was that the psychological component of the bio-psycho-social needs of participants were assessed with the BPS Risk Screener. While the tool is validated and reliable, assessment of mental health conditions such as depression, could have been more rigorous with a psychologist or psychiatrist. Based on the findings of the dimension on mental health of the EQ-5D-5L Index that remained unchanged, we believe that further research is needed on interventions that are more targeted towards mental health needs and are of a longer follow-up period to assess changes in mental health, including anxiety and depression. Another limitation was that true baseline measurements might have been overestimated, as the baseline survey was taken within a relatively long period of 60 days from enrolment date whereby some benefits of PCMH might have already taken place. Hence, our findings may be biased towards the null. Additionally, this study did not include data collection on functional ability (e.g. BADL, IADL) and future research could include these variables for a fuller understanding of patient outcomes. Lastly, patients recruited in this study were of an older age-group with bio-psycho-social needs, which may make generalisability to a more diverse population limited.

## Conclusions

This study prospectively evaluated a demonstration of a PCMH care model for older patients with complex bio-psycho-social needs. A model of care that incorporates both clinic-based and home-based health and social services, comprehensive assessments, a multidisciplinary team, individualised care plans, and shared decision-making have potential to benefit QoL in terms of needs satisfaction as well as patient activation in the long-run. The identification of high needs patients for the targeted provision of high quality care coordination and integrated care should have more emphasis in primary healthcare. Further research is needed on PCMH in various settings for a more comprehensive understanding of this model of care, and studies should be conducted using larger sample sizes, for longer durations and with randomised controlled designs, in order to draw more definitive implications and conclusions from the PCMH model of care.

## Data Availability

The datasets generated and/or analysed during the current study are not publicly available due to strict government confidentially. The corresponding author can be contacted on this matter.
